# Heart rate variability in multibacillar leprosy: Linear and nonlinear analysis

**DOI:** 10.1371/journal.pone.0180677

**Published:** 2017-07-27

**Authors:** Marcio Clementino de Souza Santos, Luiz Carlos de Lima Silveira, Sílvia Cristina Garcia Moura-Tonello, Alberto Porta, Aparecida Maria Catai, Givago da Silva Souza

**Affiliations:** 1 Pará State University, Center for Biological Sciences and Health, Belem, Pará, Brazil; 2 Federal University of Pará, Tropical Medicine Nucleus, Belem, Pará, Brazil; 3 Federal University of Pará, Institute of Biological Sciences, Belem, Pará, Brazil; 4 Ceuma University, São Luís, Maranhão, Brazil; 5 Federal University of São Carlos, Physiotherapy Department, São Carlos, São Paulo, Brazil; 6 Department of Biomedical Sciences for Health, University of Milan, Milan, Italy; 7 Department of Cardiothoracic, Vascular Anesthesia and Intensive Care, IRCCS Policlinico San Donato, Milan, Italy; Niigata Daigaku, JAPAN

## Abstract

**Objective:**

To evaluate the heart rate variability (HRV) in patients with multibacillary leprosy using dynamic linear and nonlinear analysis.

**Material and methods:**

Twenty-one leprosy patients (mean age: 39.14 ±10.58 years) and 21 healthy subjects (mean age: 36.24 ± 12.64 years) completed the sample. Heart rate variability recording was performed by a Polar RS800 CX heart monitor during a period of 15 min in the supine position and 15 min in a sitting position. Analysis of HRV was performed by frequency domain from high frequency (HF) and low frequency (LF) spectral indexes in absolute and normalized units. The nonlinear analysis of HRV was calculated using symbolic analysis (0V%, 1V%, 2LV% and 2UV% indexes), Shannon entropy (SE) and normalized complexity index (NCI).

**Results:**

*Linear analysis*: both groups showed higher HF values (p < 0.05) and smaller LF values (p < 0.05) in supine than in sitting position. The leprosy patients showed higher LF values (p < 0.05) and smaller HF values (p < 0.05) compared to the controls on supine position. *Symbolic analysis*: leprosy patients had higher 0V% values (p < 0.05), smaller 2LV% values (p < 0.05) and 2UV % values compared to healthy subjects on both positions. The 1V % had higher values (p < 0.05) for leprosy patients than for controls in the sitting position. The control subjects had smaller 0V % values (p < 0.05), and higher 2UV % values (p < 0.05) in the supine position compared to the sitting position. Leprosy patients had higher 2UV index values (p < 0.05) in the supine position compared to the sitting position. In the complexity analysis, leprosy patients had smaller SE and NCI values (p < 0.05) than the control in the supine position. There was no difference between the SE and NCI values of leprosy and the control subjects in the sitting position. The control subjects had higher SE and NCI values (p < 0.05) in the supine position than in the sitting position.

**Conclusion:**

Leprosy patients had higher sympathetic modulation and smaller vagal modulation than controls, indicating less HRV and cardiac modulation with lower complexity. The control group displayed significant HRV differences in response to position changes while leprosy patients had fewer HRV differences after the same postural change. An analysis of HRV with linear and non-linear dynamics proved to be a reliable method and promising for the investigation of autonomic dysfunction in patients with multibacillary leprosy.

## Introduction

Leprosy is a chronic, infectious disease caused by *Mycobacterium leprae* [1]. More than 200,000 new cases are detected every year, most of them in developing countries such as India and Brazil [2]. The disease leads to several disabilities, including motor, sensory, and autonomic impairments [3, 4]. These complications affect the quality of life of the patients and are associated with high treatment and rehabilitation costs.

There is a significant amount of knowledge about the impairment of the motor, sensory, and autonomic functions caused by leprosy [5, 6]. The investigation of nervous functions has been important for identifying possible techniques that monitor the neurological condition of patients, even when they have no clinical symptoms. Some studies have reported the association between peripheral neuropathy in leprosy and autonomic impairments such as cardiac autonomic neuropathy [7,8] and the impairment of vasomotor reflexes [9,10]. The altered coordination between sympathetic and parasympathetic actions of the autonomic system is prejudicial to patients suffering from leprosy due to decreased responsivity of heart function to environment or internal changes [7,11].

HRV evaluation is usually done as a function of the time or temporal frequencies from linear analysis methods [12, 13]. The decomposition of the HRV time series in the temporal frequency domain shows peaks below 0.04 Hz (very low frequency, VLF), between 0.04 and 0.15 Hz (low frequency, LF), and between 0.15–0.4 (high frequency, HF).

Alternatively to the linear methods, new methods of HRV analysis have been proposed, and non-linear methods have been applied to distinguish the autonomic contributions between the HRV modulation [14–15]. A recently introduced nonlinear approach to short-term HRV is the symbolic analysis [16]. This methodology considers the qualitative properties of the heart rate (HR) time series and is related to the complexity of specific components of HRV's being sensitive to detect nonreciprocal autonomic change, e.g. activating only with sympathetic, parasympathetic, and coactivation autonomic processes of the subsystems [16,17].

Other approach helps to separate the sympathetic and parasympathetic contribution for the HRV modulations is to associate body position changes to the HRV analysis. In healthy individuals, heart rate variability has been compared through the supine position and right and left lateral decubitus [18]; supine and sitting postures [19], supine and standing postures and supine, standing and upright and downward sloping postures [20]. In healthy subjects, the autonomic balance does not change significantly with different recurrent postures, but it is clearly different between the supine and vertical postures (standing or sitting). The sympathetic nerve function predominates in vertical postures while the vagal function predominates in reclining postures [18–20]. As there are many indications of alteration of the autonomic nervous system caused by leprosy, we hypothesized that the autonomic balance in different postures could be altered in patients suffering with the disease compared to the healthy condition.

This HRV analysis has been applied to infer the autonomic function [16–17, 21–27]. However, there are no studies in the literature comparing cardiac autonomic modulation with symbolic and complexity analyses in multibacillary individuals at rest and in response to a postural change. This form of analysis could provide additional information about the impaired cardiac autonomic function involvement in leprosy. The relevance to evaluating the HRV in leprosy patients is that this method is one more way to identify autonomic nerve functions that, together with other findings of autonomic, sensory, and motor functions, can assist in detecting nerve damage and the beginning of the treatment. Additionally, understanding sympathetic and parasympathetic heart functions can allow leprosy patients to help in the early prevention and treatment of heart diseases [28].

Therefore, the present study aimed to compare the influence of postural changes using the results of the linear (spectral) and nonlinear (symbolic and complexity) analyses of short-term HRV in multibacillary and healthy subjects.

## Materials and methods

### Ethical statement

This study complies with the declaration of Helsinki and was approved by the Ethical Committee for Research with Humans at Centro de Ciências Biológicas da Saúde of the Universidade do Estado do Pará (Report #82226). All the subjects gave written consent to participate in the study after receiving information about it.

### Subjects

Twenty-one leprosy patients (16 males and 5 females, mean age: 39.14 ± 10.58 years) diagnosed more than two years prior to enrollment and 21 healthy subjects (16 males and 5 females, mean age: 36.24 ± 12.64 years) composed the sample. The women in both groups were of reproductive age and did not use oral contraceptives. The subjects underwent a cardiovascular evaluation performed by a cardiologist that included a questionnaire about their lifestyles and clinical history, which was followed by a physical examination and a resting electrocardiogram (ECG) to confirm the presence of sinus rhythm and the absence of comorbidities. No leprosy patient or control subject performed regular physical activity. All leprosy patients had multibacillary leprosy.

### Experimental procedures

The HRV recording was conducted at the Cardiorespiratory Physiology Lab at Universidade da Amazônia, Brazil. The room temperature was artificially controlled between 22°C and 24°C, and air humidity was kept between 40% and 60%. The HRV was obtained using the heart monitor Polar RS800CX (Polar Electro Oy, Finland), which captured the R wave of ECG with a sampling rate at 500 Hz. The temporal distance between two consecutive R-wave peaks was taken as RR interval (RRi). The series was computed by the software Polar ProTrainer 5 (Polar Electro Oy, Finland) and exported for further custom-developed analysis.

The volunteers lied down for 10 minutes and were not allowed to talk or perform movements to stabilize the cardiovascular parameter. Afterward, the heart rate capture was performed for 15 minutes in the supine position. Then, the subject moved from the supine position to the sitting position with a backrest with the knees flexed at 90° and the feet touching the floor. Again, the heart rate was recorded during 15 minutes. All the data were collected in the same time range (between 8:00 a.m. and 12:00 a.m.). Linear and nonlinear analysis were carried out using software developed by A.P [29].

### HRV analysis

#### Signal correction and selection of sequence

RRi series were visually inspected searching for possible outliers corresponding to missing R-wave detections, arrhythmic epochs or erroneous R-wave delineations. Only period free of evident outliers was selected. In agreement with the indications of short-term HRV analysis [30] we selected series of 256 beats with greatest stability, for each subject, in a random position inside each studied condition (rest and sitting) to standardize the analysis. The choice of 256 RRs instead of the more usual length of 300 [30] is to speed up analysis based on Fourier transformation and presented in previous clinical investigations [14,31–36]]. The stationarity of the sequence was tested according to Magagnin et al. [37].

#### Linear analysis

The power spectrum of RRi series was computed with a Fast Fourier Transform to separate the frequency components from the time series. The total power (ms2) was estimated from high frequency (HF: 0.15–0.4 Hz), low frequency (LF: 0.04–0.15 Hz), and very low frequency (0.003–0.04 Hz). HF and LF powers were normalized by the subtraction of the VLF from the total power shown in Eqs 1 and 2.

$$\text{HF }\left(\text{nu}\right)\;=\;100\;\times \;\frac{\text{HF}}{\left(\text{total power }–\text{ VLF}\right)},$$ (1)

$$\text{LF }\left(\text{nu}\right)\;=\;100\;\times \;\frac{\text{LF}}{\left(\text{total power }–\text{ VLF}\right)},$$ (2)

LF is the low-frequency power, HF is the high-frequency power, VLF is very low power, nu is normalized units, and total power is the integrated power divided by the variance in the observed spectrum between the edges of the VLF (lower edge) and HF (higher edge) [12,23,38].

#### Nonlinear analysis: Symbolic analysis

We performed the quantization of the HRV series into a sequence of integers or symbols, as described by Porta et al. (2001) [25]. The RR series was spread over 6 symbols with the resolution of the differences between the maximum and minimum of the series divided by six (number of symbols). The quantization procedure transformed the RR series into a sequence of integers between 0 and 5. Each sequence of three integers was considered a pattern. It was possible to find 216 different patterns. The patterns were grouped into four families: 0V, 1V, 2LV, and 2UV. The 0V group included sequences of three equal symbols, such 111. The 1V group included sequences of two consecutive equal symbols and one different symbol, for example, 221 or 355. The 2LV group included sequences of three symbols in ascending or descending order, such as 035 or 421. The 2UV group included sequences of three symbols where the one in the middle was lower or higher than the two other symbols, for example, 132 or 523. We calculated the rate of occurrence of these families (0V%, 1V%, 2LV% and 2UV%).

#### Nonlinear analysis: Shannon entropy (SE) and conditional entropy (CE)

We estimated the SE as Porta et al. (2001) do [25]. We took the SE as an indicator of the complexity of the distribution of the patterns. The higher the number of patterns, the higher the SE. If a specific pattern dominated the distribution, the SE would be lower.

The CE measures the information carried by the most recent value that cannot be resolved from past samples. The higher the predictability, the higher the regularity and the lower the complexity of the sample. We followed the approach proposed by Porta et al. (1998) [16] that estimates CE after uniformly partitioning the multidimensional embedding space into non-overlapping cells, estimating the probability of the patterns necessary to compute the CE. Since the CE estimate is biased and this bias is particularly evident in the case of short sequences of data, Porta et al. [16] proposed to correct the CE estimate with a corrective term, thus defining the so-called corrected CE (CCE). When the CCE is plotted as a function of the number of previous samples utilized to condition the behavior of the current value, it exhibits a minimum if repetitive patterns are present in the series. The minimum of the CCE normalized by the SE is taken as an index of dynamical complexity of the series [27] and is hereafter denoted as normalized complexity index (NCI). The NCI ranges from 0 (null information) to 1 (maximum information) [27].

### Statistics

All parameters estimated in the present study had normal distributions evaluated by the D'Agostino Pearson test. We used two-way repeated measure analyses of variance (Bonferroni posthoc test for multiple comparisons). The significance level for all analyses was 5%. We used the software Biostat 5.0 [39].

## Results

Table 1 describes the physical features and information about the drug therapy of the leprosy patients.

**Table 1:** Values of the age, anthropometric characteristics, drug therapy and dosage of medicine in leprosy group volunteers.

LeprosyGroup	Age	Body Mass (Kg)	Height (m)	BMI (Kg/m^2^)	Drug Therapy	Dosage (mg)
**1**	42	65	1.7	22.49	Prednisone	30
**2**	36	63	1.65	23.16	Prednisone	20
**3**	52	72.5	1.7	25.08	Prednisone	50
**4**	53	74	1.72	25.08	Prednisone	20
**5**	35	70.3	1.7	24.32	Prednisone + MDT	30
**6**	23	79	1.7	27.33	Prednisone	20
**7**	55	65	1.65	23.89	Prednisone	30
**8**	54	57	1.62	21.75	Nd	-
**9**	40	85.7	1.63	32.33	Prednisone	5
**10**	36	71	1.74	23.5	Prednisone	60
**11**	34	81	1.78	25.63	Prednisone + Thalidomide	20 + 100
**12**	55	62	1.65	22.79	Prednisone	20
**13**	26	46	1.55	19.16	Prednisone	5
**14**	29	63	1.75	20.58	Prednisone	5
**15**	26	67	1.65	24.63	Prednisone	5
**16**	42	76	1.71	26.02	Nd	-
**17**	41	63.2	1.65	23.23	Prednisone	70
**18**	38	56	1.51	24.56	Prednisone	20
**19**	47	70	1.7	24.22	Prednisone	5
**20**	22	62	1.52	26.83	Nd	-
**21**	36	78	1.78	24.68	Nd	-
**Mean**	**39.14**	**66.76**	**1.67**	**24.35**		**24.69**
**SD**	**10.58**	**9.24**	**0.08**	**2.66**		**20.12**

MDT, Multi droug therapy; nd, no drugs. SD, standard deviation

The anthropometric features of the volunteers from both groups are shown in the Table 2. There was no significant difference between the anthropometric measurements estimated from healthy and leprosy groups.

**Table 2:** Anthropometric characteristics of the volunteer groups.

Characteristics	Control Group	Leprosy Group	P-value
**Age (years)**	36.24 ± 12.64	39.14 ± 10.58	0.212
**Weight (kg)**	75.79 ± 10.62	66.76 ± 9.24	0.058
**Height (m)**	1.73 ± 0.09	1.67 ± 0.08	0.063
**BMI (kg/m2)**	25.45 ± 2.84	24.35 ± 2.66	0.100

Data are reported as means ± standard deviation. BMI = body mass index

### HRV time domain and spectral analysis

The RRi means of the volunteers from both groups are shown in the Table 3. The RRi means were higher in the supine position than in the sitting position (p < 0.05) for both groups. The intergroup comparison showed that the leprosy group had lower RRi values in the sitting position (p < 0.05).

**Table 3:** R-R intervals (ms) values of the groups.

	R-R interval (ms)	
Position	CG	LG
**Supine**	870 ± 122	848 ± 143
**Sitting**	843 ± 106*	796 ±137*†

Data are reported as means ± standard deviation of RRi (ms), CG: control group; LG: leprosy group.

* p<0,05 compared to the supine position.

^†^ p<0,05 compared to the same position in the control group.

**Fig 1:**
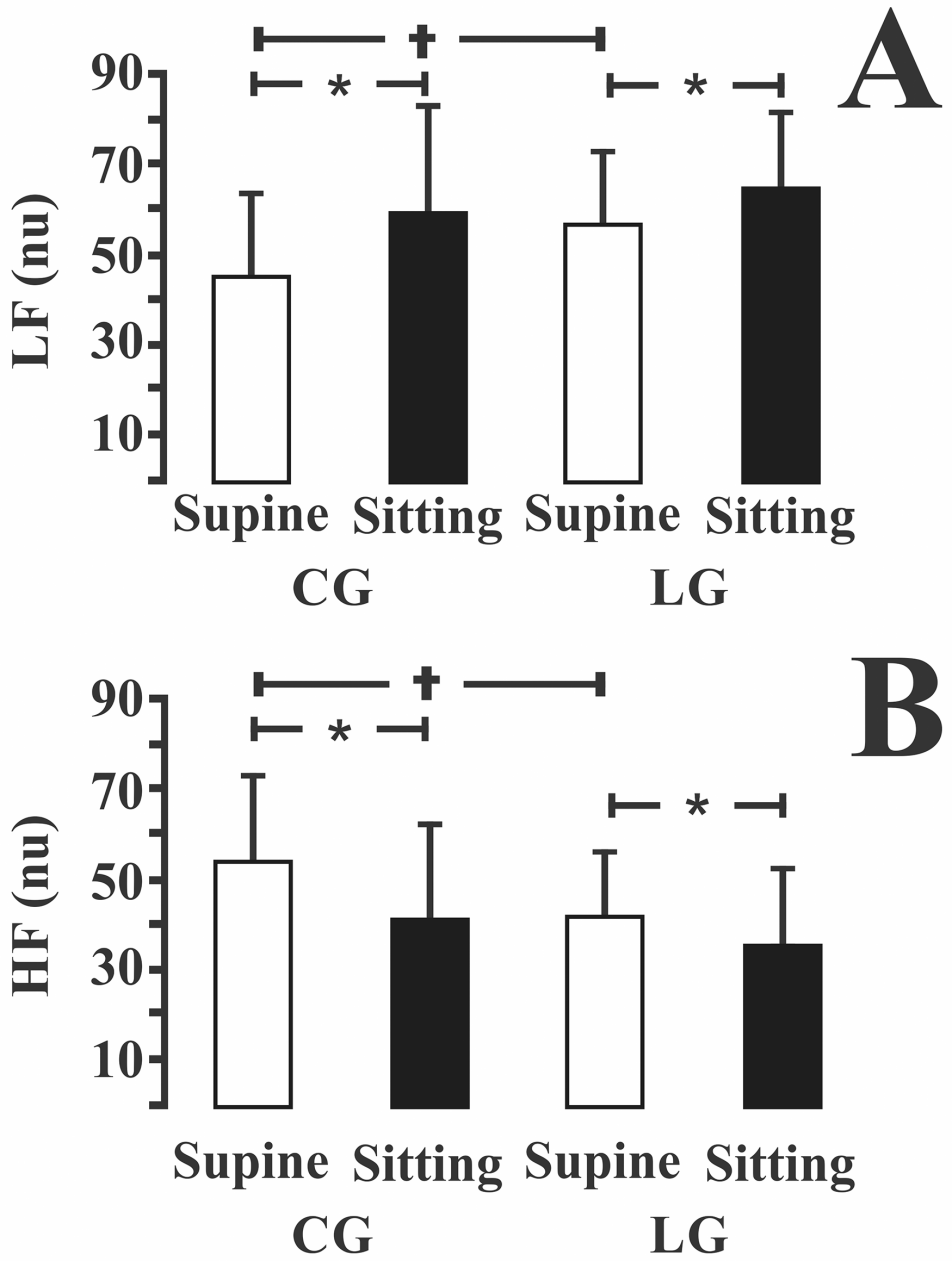
Linear analysis of the HRV from control group and leprosy groups for (A) low frequencies and (B) high frequencies in normalized units (nu). Leprosy patients had higher LFnu values (lower HFnu values) than controls in the supine position. Both groups were influenced by the change from supine to sitting position. LFnu values increased and HFnu values decreased in the sitting position compared to supine position for both groups. * p<0.05 intragroup; † p<0.05 intergroup.

Fig 1 shows a comparison between the linear analysis of data estimated from the control group (CG) and the leprosy group (LG). Intergroup differences were observed for LFnu and HFnu values in the supine position (p < 0.05, Fig 1A) but not in the sitting position (p > 0.05). In the supine position, the control subjects had lower LFnu values and higher HFnu values than the leprosy group. Both the control subjects and leprosy patients were influenced by the postural change. Both groups displayed significant increases in LF values and decreases in HF values in the sitting position (p < 0.05).

### Symbolic analysis

**Fig 2:**
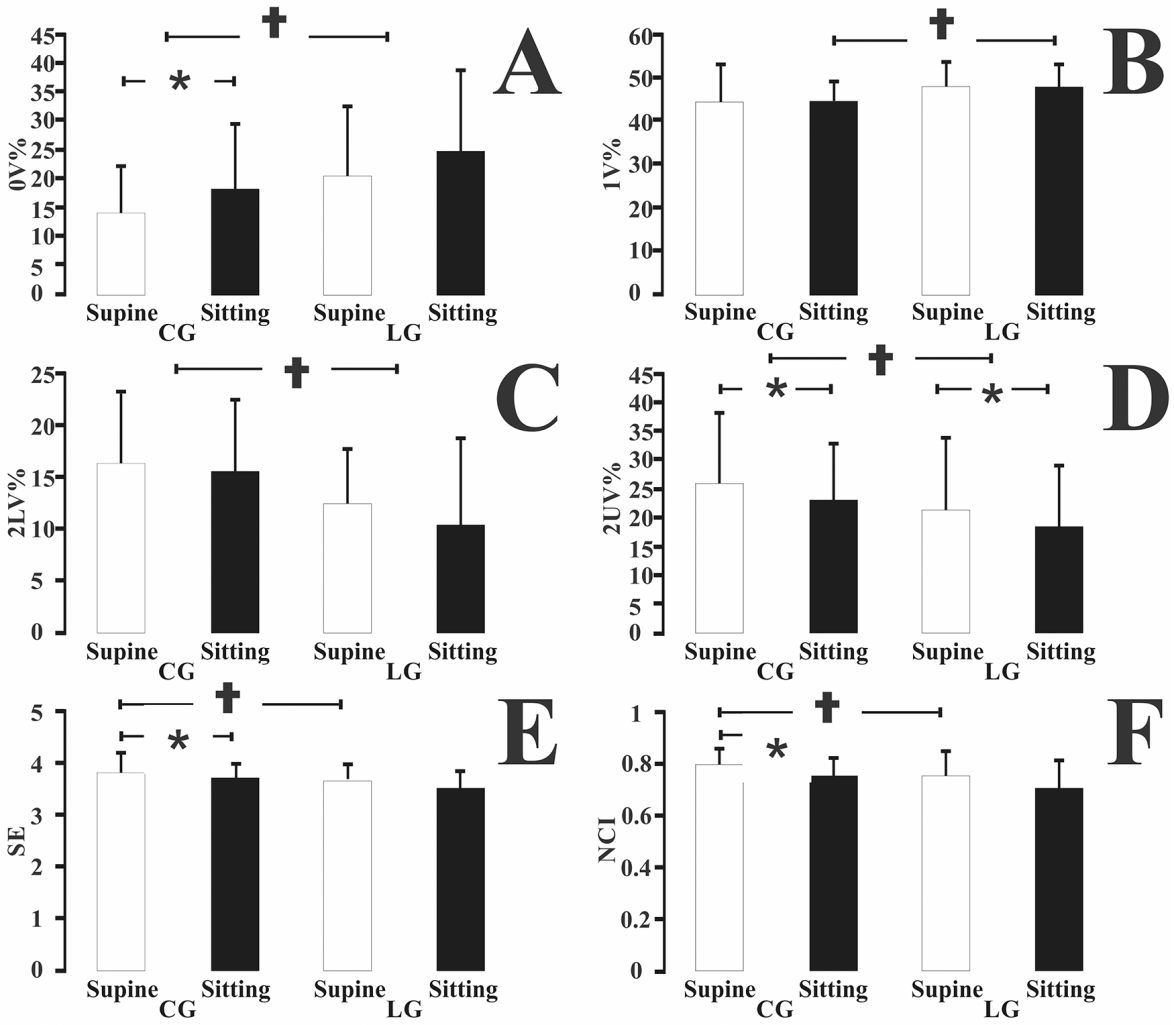
Non-linear analysis of the HRV for (A) 0V%, (B) 1V%, (C) 2LV%, (D) 2UV%, (E) Shannon entropy, and (F) NCI. All the indexes showed differences between control and leprosy groups. The leprosy group in most of the indexes showed no differences between the sitting and supine positions. * p<0.05 intragroup; † p<0.05 intergroup.

Fig 2 shows a comparison between the symbolic analysis of the two groups. We observed intergroup differences in the four families of patterns. For the supine position, we observed that the leprosy group had higher 0V% values and lower 2LV% and 2UV% values than the control group (p < 0.05). For the sitting position, we found that the leprosy group had higher 0V% and 1V% values and lower 2LV% and 2UV% values than the control subjects. The postural change from the supine to the sitting position significantly increased 0V% values in the control group and decreased the 2UV% values in both groups.

For the supine position measurements, the leprosy group had lower SE and NCI values than the control group (p < 0.05). There was no intergroup difference between the SE and NCI values. In the change from the supine to the sitting posture, we observed that only the control group had a significant change in the SE and NCI values (p < 0.05).

## Discussion

In the present investigation, we observed that multibacillary leprosy patients had changes in HRV using linear and non-linear methods, reflecting changes in the autonomic control of the heart function. We found in the patients an increase of the sympathetic modulation and a decrease of the vagal modulation on the heart as well as a reduced complexity of HRV.

HRV represents a reliable method which to evaluate the autonomic system [40]. Linear and non-linear methods of HRV analysis have been associated with the activation of the sympathetic and parasympathetic nervous systems [41]. Both methods of analysis have shown changes in the HRV across the aging process [17] due to heart disease [42] and diabetes [14].

Direct entry to terminal nerves and vascular and lymphatics pathways to the cells infection are probably the main routes of the invasion of *M*. *leprae* in the tissues [43]. The mechanism that underlies the nerve damage in leprosy is still unclear. *M*. *leprae* has tropism for Schwann cells, which envelop the axons. An inflammatory response to lyse the infected Schwann cells occurs after the Schwann cells presents *M*. *leprae* antigens to T cells, which is followed by damage to the nerve associated with the glia [44]. Evidence has shown that the leprosy bacteria reprograms the genetic code of Schwann cells, turning them into immature migratory cells that could be turned into other types of cells. This novel mechanism would allow the spread of the bacteria to other tissues in the body [45].

The autonomic involvement of leprosy was first described by the presence of the bacteria in the sympathetic chain and vagus nerves [46,47]. Later, several studies reported autonomic changes in leprosy patients such as focal anhidrosis, syncope, gustatory sweating, erectile dysfunction, impaired cutaneous and muscle vasomotor reflexes, and cardiac autonomic function [11,48–52].

The control of the cardiac function is altered in leprosy due to the infection itself, the drug therapy, the systemic changes that the disease can evoke, and the loss of autonomic innervation [9]. The monitoring of the heart activity became an important indicator of the autonomic nervous system's condition. Ulvi and colleagues [11] and Soysal and colleagues [7] investigated the variation of R-R intervals in leprosy patients. Ulvi et al. evaluated 37 lepromatous leprosy patients, and Soysal et al. evaluated 29 leprosy patients. Both studies evaluated RRi from electromyographs with specific frequencies and sensitivity for QRS complexes; they captured the variations in time between them and observed that the leprosy patients had smaller variations in RRi. The authors of both studies interpreted the low HRV of the leprosy patients as an indication of a higher sympathetic modulation and a lower vagal modulation of the heart [7, 11]. However, this is not necessarily correct because it is hard to state whether a low HRV is caused by a lower parasympathetic modulation, a higher sympathetic modulation, or both when only RRi are considered.

Our study investigated the RRi from leprosy patients in the two positions for 15 minutes. We applied linear and non-linear methods in the same series of 256 points for both conditions. Similarly to Soysal et al. and Ulvi et al. [7, 11], the linear analysis in the present study showed that leprosy patients in the supine position had lower values for HFun and higher values for LFun than healthy subjects. The interpretation of these results is not easy because it is known that the LF band is not predominantly sympathetic, a phenomenon thoroughly investigated in several studies [53–56]. Other methods and analyses should be studied regarding the linear analysis of the HRV to arrive at a clearer interpretation of the results. After the postural change, both groups experienced increases in LFnu power and decreases in HFnu power.

Linear methods allow an interpretation of the system as a whole (reductionist). However, most natural systems display nonlinear behavior, and the cardiac system is not different. From these nonlinearities arise chaotic and complex behaviors with fractal characteristics, which enable the human body to adapt to different environments [57].

Porta et al. [16] proposed the non linear analysis of short term heart period variability, whose patterns are classified and divided into four families. The non linear analysis can detect nonreciprocal autonomic change [16,24,58]. We performed the first non-linear analysis of HRV in leprosy patients. The non-linear analysis by symbolic analysis indicated higher sympathetic activation in the leprosy patients, indicating a lower HRV. The leprosy patients had high 0V% values and low 2LV% and 2UV% values in both positions. The 1V% was high in the leprosy patients only in the sitting positions. The 0V% index is associated with sympathetic modulation while the 1V% indicates a simultaneous modulation of the vagal and sympathetic systems. The 2LV% and 2UV% indexes are associated with vagal modulation [15,24,25]. The leprosy of the multibacillary type is characterized by important nerve damage and autonomic neuropathy like in diabetic patients [14,59].

The analysis of complexity also was a differential in our investigation. The Shannon entropy (SE) and normalized conditional entropy (NCI) of the leprosy HRV data were lower than for the controls. Per definitions, the SE is a measure of the complexity of the pattern distribution while conditional entropy provides information about the complexity of the dynamic relationship between one pattern and the next one (regularity) [16]. A significant increase in regularity (i.e., a decrease in NCI) was observed in the multibacillary leprosy group compared to healthy groups, thus demonstrating that patterns formed more regular and predictable sequences, which decreased NCI. Additionally, the complexity of heart period dynamics depends on the autonomic nervous system, i.e., it decreases in the presence of increased sympathetic modulation [27].

Our findings indicate that the multibacillary leprosy patients have a lower complexity because of alterations of the cardiac autonomic modulation with sympathetic predominance in supine. It is noteworthy that the patients investigated in our study had no clinical symptoms of heart disease. The HRV evaluation had enough sensitivity to show alterations in the heart function; previously, the patients had been diagnosed some cardiac disease.

In relation to postural change, we observed that the control group had significant differences that were not observed in the leprosy group for the 0V% index. The leprosy group showed differences between the sitting and supine position only for the 2UV% index. For SE and NCI, we also observed that only the control group had significant differences when comparing the sitting and supine position results. These results indicate that the control group has a greater predominance of vagal modulation, a greater HRV, and a greater complexity of cardiac autonomic modulation [16,24]. The change of position from the supine to the sitting position represents a stimulus, especially for the sympathetic modulation [14,16,24,58,60]. In the supine position, both autonomic systems have tonic activity with vagal modulation predominance that caused a high HRV while the sitting position had a sympathetic modulation prevalence and led to the decrease of the HRV [37,40]. The leprosy group presented more sympathetic modulation on the heart in the supine position. This may have contributed to the slight difference observed in postural change since the already sympathetic modulation increased in the supine condition. Similar results were found for diabetic patients [50,58,59].

In the present study, we evaluated only multibacillary patients because they are a sample with high sensitivity and motor damage. They are characterized by diffused infiltration of the *Mycobacterium leprae* in the tissue, multiple lesions with symmetric distribution in multiple nerves, and a loss of sensory and motor function, similarly to diabetic neuropathy [61].

The use of the linear and non-linear analysis of the HRV for the periodic evaluation of leprosy patients can be helpful in the early diagnosis of the heart function control. The early diagnosis of that would help apply interventions in order to avoid more functional complications.

## Conclusion

The leprosy group had higher cardiac sympathetic modulation, reduced vagal modulation on the heart and reduced complexity of RRi than the control group, indicating lower HRV. In response to postural change from supine to sitting position, the leprosy group showed a lower change to this stimulus because even in the supine it already had high sympathetic modulation.

The HRV analysis by linear and nonlinear dynamics proved to be a sensitive and promising method for investigation of autonomic dysfunction in patients with multibacillary leprosy.

## Supporting information

S1 File Database for leprosy and control group. (XLSX)
